# Limited knowledge of health risks along the illegal wild meat value chain in the Nairobi Metropolitan Area (NMA)

**DOI:** 10.1371/journal.pone.0316596

**Published:** 2025-03-26

**Authors:** Sherril Phyllis Masudi, James Hassell, Elizabeth AnneJessie Cook, Pim van Hooft, Frank van Langevelde, Ralph Buij, Moses Yongo Otiende, Joel Winyo Ochieng, Andrea Santangeli, Anise Happi, Samuel Nsikan Akpan, Lian Francesca Thomas

**Affiliations:** 1 Wageningen University and Research, Wageningen, Netherlands; 2 International Livestock Research Institute, Nairobi, Kenya; 3 Smithsonian’s National Zoo and Conservation Biology Institute, Washington DC, United States of America; 4 Animal Ecology Group, Wageningen Environmental Research, Wageningen, Netherlands; 5 Wildlife research and Training Institute, Naivasha, Kenya; 6 University of Nairobi, Faculty of Veterinary Medicine, Nairobi, Kenya; 7 Animal Demography and Ecology Unit, Institute for Mediterranean Studies (IMEDEA), Esporles, Spain; 8 African Center of Excellence for Genomics of Infectious Diseases, Ede, Nigeria; 9 Royal (Dick) School of Veterinary Studies, University of Edinburgh, Midlothian, United Kingdom; University of British Columbia, CANADA

## Abstract

Consumption of and trade in wild meat could result in infectious pathogen spillover into human populations. Such spillovers could propagate into sustained outbreaks in major cities where human aggregations potentially catalyze their spread. A better understanding of how urban wild meat value chains operate could assist in mitigating spillover events. We used key informant interviews and literature review to understand the structure and operations, actors, their practices, and health risk perceptions along a wild meat value chain supplying a rapidly urbanizing city in Africa, the Nairobi Metropolitan Area (NMA). The value chain operates via three main nodes: harvester, trader, and consumer nodes. We found wild meat to be harvested from peri-urban areas of the NMA, consumed or sold locally, or supplied to distant urban markets. Actors reported increased participation along the value chain during the dry season, and over the Christmas period. The value chain operated informally, creating a ‘rules in use’ framework focusing on sanction avoidance, while ignoring food safety concerns. Consequently, respondents reported slaughtering wild animals on the bare ground, handling wild meat with unwashed hands and uncleaned utensils. No value chain actors reported wearing personal protective equipment when handling wild meat. At the distant markets’ trader node where wild meat was sold as livestock meat, meat vendors engaged in similar unsafe practices. Actors had limited awareness of the specific health risks from wild meat. We speculate that the observed limited health risk awareness, and sanction avoidance attempts promotes unsafe practices during exploitation of wild animals for food, income and for medicinal purposes. Multisectoral efforts at the conservation and public health nexus, as well as community education on the potential health risks from wild meat are key in reducing potential spillovers.

## Introduction

Wild meat is a major source of livelihoods and nutrition in the tropics, yet also a potential source of infectious diseases [1–4]. Wild meat, also known as game or bushmeat, refers to any fat, flesh, blood, or any other tissue of wild animals that could be either fresh or processed, and is intended for consumption, sale or other uses [5,6]. Wild animals are hunted in the rural and peri urban areas and their meat is supplied through informal [7–9], sometimes illegal [10] value chains for sale and consumption. Supply is either locally at hunting sites, or to more distant urban centers [11,12] including international markets [13,14]. However, human interaction with wild animals and wild meat along these value chains creates a route by which the value chain actors and urban dwellers alike can be exposed to wild meat-borne pathogens; zoonotic and foodborne [8,15,16]. Zoonotic pathogens circulating within wild animal populations have a potential for spillover into human populations [3]. On the other hand, foodborne pathogens can be introduced into wild meat either from the animals themselves or via cross-contamination from unhygienic handling [15,17,18]. A better understanding of how urban wild meat value chains operate and the processes that could drive human exposure to wild meat-borne pathogens can assist surveillance and mitigation of potential spillover threats [8,19].

The risk of human exposure to infectious pathogens can occur at different points along the wild meat value chain, such as during harvesting, processing, sale, or consumption [15,20]. Hunters and poachers could be exposed to pathogens during harvesting and initial processing [8]. Wholesalers and retailers could be exposed as they prepare wild meat for display and sale. Lastly, consumers could be exposed to pathogens during wild meat preparation and consumption, commonly at home, or at commercial eateries. The risk of actors’ exposure to infectious pathogens is further dependent on actors’ food safety practices. There is extensive documentation showing that wild meat handlers often do not comply with food safety and hygiene practices [8,16,21,22]. Additionally, the legal laws against wild meat harvesting, consumption and sale also trigger covert harvesting, trade, and consumption of wild meat, with generally complete disregard for food safety considerations [23–25]. Consequently, previous spillover events have been attributed to unsafe wild meat handling practices [3].

The risk of infectious pathogens spillover along wild meat value chain extends spatially from the wild animal habitats to local homes, and to terminal urban and peri-urban markets. The risk could extend even across transnational boundaries via wild meat export [13]. Given the differing risk profiles for pathogen spillover along the value chain, using a value chain approach to map the risk factors for actors’ exposure to pathogens is crucial. Most studies of wild meat value chains, however, focus only on addressing their biodiversity impact [[7,10,26,27]]. It is only recently that efforts have begun to recognize the importance of using a value chain approach to address the risk of infections and disease emergence from wild meat trade and consumption [16,28,29]. Even so, the recent efforts are yet to focus extensively on major urban centers, particularly in Sub-Saharan Africa. Supply of wild meat to urban centers could facilitate the emergence and resurgence of pathogens within susceptible populations where rapid pathogen transmission and amplification could have adverse impacts [30]. This is particularly important for the Nairobi Metropolitan Area (NMA), a rapidly urbanizing city region where covert harvesting, consumption and sale of wild meat has been reported [31–33]. In its covert nature, any spillover events from the wild meat could rapidly amplify and spread within large populations, including across international borders. However, there has not been any dedicated studies to document the wild meat value chain in Kenya [14], and its potential health implications.

For this study, we used a qualitative study approach based on key informant interviews and a literature review to understand the potential risk factors for pathogen exposure and infection along the wild meat value chain operating in the NMA. We addressed the following research inquiries; What regulatory framework does the wild meat value chain in the NMA operate on? Which actors are involved in the value chain operations? What are the actors’ roles, practices, and perceptions regarding the health risks from wild meat? Which wild animal species do they target? Are there temporal and spatial factors that influence the value chain’s operation? Data from our study is vital for surveillance and mitigation of potential infectious pathogen spillover from wild meat consumed and traded in the NMA.

## Materials and methods

### Ethical approvals and other considerations

This study was approved by the Institution Research and Ethics Compliance (IREC) at the International Livestock Research Institute (ILRI, Kenya) and the National Commission for Science, Technology, and Innovation (NACOSTI, Kenya). All protocols, as outlined in the ILRI-IREC2022-39 and NACOSTI/P/23/23263 research permits were adhered to. Before every interview, we shared the project information with the participants through a Project Information Sheet – PIS (S1) and sought their consent to participate in the study. The project information sheet also contained detailed information on how we planned to protect any study participants from any legal reparations based on the data they shared with us. All participants gave a verbal consent to participate, and this was witnessed by the lead author.

Due to the sensitivity of this study, measures were put in place to ensure the protection of our study participants’ privacy and confidentiality. During community entry and engagement, we intentionally excluded Kenya Wildlife Services (KWS) personnel to allay fears of recrimination on participants. Additionally, we chose to use a qualitative approach of data collection as based on previouslydocumented decision tree protocol for sensitive studies [34], The protocol outlines data collection approaches for complex conservation challenges such as poaching and illegal wild meat trade. The decision tree provides alternative questioning methods that would ensure participants privacy while still encouraging reporting of sensitive data. We gave the participants an option for giving verbal consent to avoid having any records of their personal data. Also, this enabled us to avoid leaving behind any traceable physical document containing the project details, which can be used to identify the study participants. We advised our study participants not to mention their names or personal identifying features during the interviews, whether recorded or taken down as notes. Where these were mentioned, the lead author coded and anonymized such data. During the interviews, participants were only limited to the lead author as the enumerator and only one respondent at a time to enhance privacy and confidentiality. Where translation was needed, the community focal person was included.

### Study area and data collection

**Fig 1:**
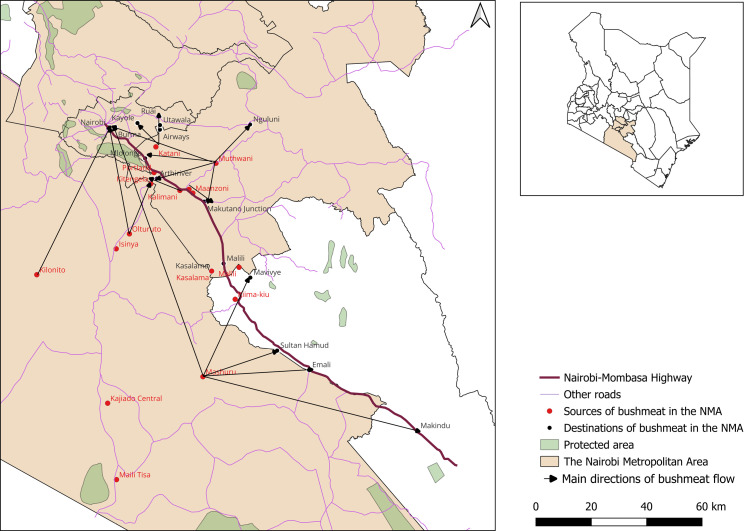
Map of the Nairobi Metropolitan Area showing the specific study sites that were identified as hotspots for wild meat harvesting, trade, and consumption. This map was produced using the open source QGIS software version 3.32 [43]. The administrative boundaries data was freely obtained from the DIVA-GIS website [44].

We conducted our study within the NMA, which refers to the Nairobi City and its surrounding counties including Kajiado, Machakos, Kiambu and Murang’a Counties [35] (Fig 1). The NMA is among the fastest growing urban regions in Sub-Saharan Africa [36], with the most recent population census size estimate at over nine million [37]. It sits on a biodiverse landscape of global importance with vast wildlife corridors and protected areas in Kajiado and Machakos Counties. Several areas in the NMA have been marked as hotspots for poaching, trade, and consumption of wild meat [38]. These formed our study sites (Fig 1).

Hereafter, we use the term “local” (local supply, sale, or markets) to refer to intentional harvesting of wild meat for food, perceived health benefits, or for sale and consumption within the local communities at poaching hotspots. Wild meat in the local value chain does not therefore enter any established premises for sale but only circulates directly among these actors. We use the term “distant” (distant supply, sale, or markets) to refer to harvesting, transport to, and sale of wild meat, raw or processed, through established premises to customers in Nairobi City County and other peri-urban markets within the NMA [39,12]. The sale of wild meat in the distant market may be disguised as meat from livestock to “unwitting” customers [40–42]. We acknowledge that distant markets for wild meat from Kenya extends internationally [13,14] and in such cases, its utilization, as medicine or food, could be intentional, just like the case of local supply. However, this aspect did not come up as a theme in this current study.

We employed two primary data collection methods: (i) Key informant interviews using an open-ended questionnaire guide, and (ii) a literature review on knowledge, attitudes, and practices related to food safety and perception among meat vendors in the NMA.

#### Key informant interviews.

Initially, we worked with KWS to design a conceptual value chain framework, through which we identified potential study sites. We defined a value chain node as the step towards creation of a product’s value and its distribution to the end users for use and disposal [19,45]. Value chain actors therefore were people involved in adding value to a product at each of the steps towards utilization and disposal of the final product, in this case wild meat. We considered three main value chain nodes (with the respective actors) during conceptualization: the harvesting node (poachers), the trader node (transporters, wholesalers, and retailers) and consumption node (consumers).

Thereafter, we recruited community focal persons from our previous research networks and personal contacts at the selected sites. Due to the sensitivity of this study, we cannot give more specific details about this process for recruiting the focal persons. The focal persons helped in the establishment of the initial contact with the communities. We used snowballing technique [46] to non-randomly identify and select our study participants. Snowballing approach was necessary because our study involved a sensitive topic on an illegal practice, thus rendering our study subjects a hard-to-reach or ‘hidden’ population [47]. As such we anticipated scantiness of information and higher refusal rates if actors were approached without referrals. We acknowledge the shortcomings of using the snowball approach and we implemented measures to mitigate them [47,48]. For instance, we had measures to protect our study participants from any legal reparations and assured them of these through the PIS (S1). Secondly, we used community focal persons drawn from within the participating populations, thus part of their social network. The community focal people were also interviewed. Lastly, the key informant interviews were conducted by the lead author, a female Kenyan PhD candidate, who had a good understanding of the communities at the study sites based on previous research partnerships. Snowballing approach has been successfully used to map value chains in other studies [26,49]. Referrals from the community focal persons and the participants also ensured that the selected participants were typical of the actor groupings along the wild meat value chain in the NMA [26,48]. Participant recruitment was conducted simultaneously with the survey in April and May 2023. Only retailers in the local market were recruited at the trader node. Data on retailers and wholesalers in the distant market were obtained from the poachers who supplied them, from actors who understood how the distant markets operate. We also obtained data on the traded node from the systematic literature review as outlined further in the next section.

In addition to the decision tool on conducting sensitive studies [34], we selected a qualitative approach to give a better understanding of actors’ practices and perceptions by capturing their personal accounts of events commonly missed in structured questionnaires [50]. We developed a key informant interview guide (S2) as based on a previous study [16]. We sought input from one of the lead authors (LFT) with expertise in designing and conducting qualitative studies and another author (AS) who is a social scientist with expertise in conducting studies involving sensitive and illegal topics.

All interviews were conducted in Kiswahili, except for two interviews that were done in the local language with a translator present. At every study site, we recruited participants until we were not able to get any new meaningful information from additional participants. We considered this as the point of data saturation [51].

#### Systematic literature review: knowledge of, attitude and practices towards meat hygiene and safety by meat handlers in the NMA.

The distant market trader node was an important node, yet one for which it was difficult to collect primary data via key informant interviews for two reasons. First, based on preliminary data provided by the KWS and findings from previous studies, it was understood that wild meat in distant markets were handled hidden, and sold disguised as, or mixed with meat from livestock [19,40,42]. Consequently, similar food safety practices, knowledge and attitudes for meat from domestic animals were applied to wild meat. Secondly, we feared that retailers and wholesalers who knowingly sell wild meat in distant markets would not agree to participate in the research. We attributed our reasoning to them fearing the known illegality of the practice that could have negative outcomes on their businesses. Indeed, poachers who supplied them would not refer us to their customers as such was ‘bad for business’. We therefore used systematic literature review (SLR) to obtain data on actor health risk practices at the distant market trader node.

A review was conducted targeting studies on food safety and hygiene practices amongst meat vendors in the NMA. This approach was used to understand practices and handling of meat in the terminal node of the distant market where wild meat might be fraudulently or illegally sold disguised as livestock meat to unwitting customers. We aimed to understand the potential for vendors’ exposure to pathogens and contamination of meat at this node.

#### Search methods, inclusion, and exclusion criteria.

**Fig 2:**
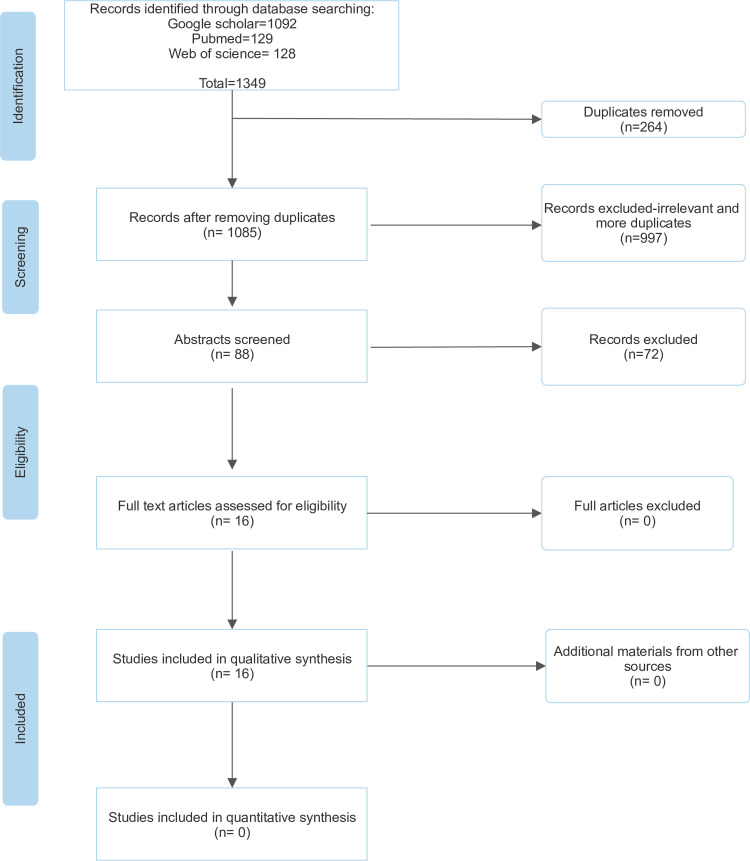
PRISMA flow chart showing the selection process in the systematic literature review of knowledge, attitudes, and practices of meat retailers towards health risks associated with handling and consumption of meat in Nairobi Metropolitan Area.

The literature review was conducted as per the PRISMA-P guidelines for conducting systematic literature reviews (Fig 2). Our PRISMA-P checklist can be found as supporting material (S3). The protocol for this review is registered with INPLASY (INPLASY202460077). The search was conducted in Web of Science, PubMed, and Google Scholar. We found it effective to use Google Scholar because it could yield any material related to the search including theses, project reports and presentation abstracts. It was important to consider these grey sources as most studies reporting on our review topic were commonly available in such forms.

We used the Boolean operators “AND” and “OR” to combine the relevant search terms. In Google Scholar, the following simple search term was used: Hygiene practices OR Food safety practices AND Meat AND Nairobi. In PubMed and Web of Science, we used the following syntax: (“Food safety practices” OR “Food hygiene practices”) AND (knowledge OR awareness OR understanding) AND (attitude OR perception OR opinion) AND (meat handlers OR butcheries OR meat vendors OR meat markets) AND (Nairobi OR “Nairobi City” OR “Nairobi metropolitan area”).

We sought data on food safety and hygiene practices and the associated knowledge and attitudes amongst meat handlers and vendors in the NMA, in the period from 2013 to 2023. We aimed to answer the following two questions from the systematic literature review: i) What food safety and hygiene practices do meat vendors in NMA engage in? ii) What knowledge, perceptions and attitudes do meat vendors in the NMA have about safe meat handling practices and the associated risks? We excluded studies discussing food handling practices but not directly relating to meat vendors, and studies reporting on meat vendors in urban centers in Kenya, East Africa or developing countries but not specific to the NMA. We included studies that were conducted in informal premises such as makeshift stalls as seen with street vendors and those that operate formally such as hotels, catering units, and meat processing companies. Studies reviewed focused on meat and meat products that were raw, cooked, or processed.

The key elements of the review questions were simplified by the PICOT acronym considering meat handlers and vendors as the population (P) but without control (C); actors’ knowledge and perceptions regarding food safety risks as interventions (I); food hygiene and safety practices as the outcomes (O) and a time frame from 2013-2023 (T). We used guidelines from the Newcastle-Ottawa scale [52] to assess individual study quality. Quality indicators, including selection criteria for study population, study comparability and assessment of the study outcomes based on the reported statistical analysis methods were used. For qualitative data (that are not considered in the outcome assessment section of Newcastle-Ottawa scale), a star (*) was awarded when we thought that the analysis protocol was exhaustively described as per the protocol by Alarcon et al. [53]. The quality assessment outcome can be found in SI5. Due to the low number of studies considered for review, only the lead author conducted the inclusion and exclusion process, data extraction and quality assessment.

### Data management and analysis

Responses from the key informant interviews were recorded in audio format and later translated to English and transcribed. When no consent was given for audio capture and responses consequently were taken down as notes, they were translated and transcribed in real time. All raw data from transcripts were anonymized and stored in a password protected computer. Anonymized transcripts were then used for data extraction. We employed a coding frame designed from the research questions to generate the following data categories.

1. Product flow, actors, and interactions along the value chain.
2. Wild animal species poached, traded, and consumed.
3. Spatial and temporal characteristics of the wild meat value chain.
4. Governance along the wild meat value chain.
5. Actors’ practices regarding the health risks associated with wild meat.
6. Actors’ knowledge of, and experience with health risks associated with wild meat.
7. Actor’s attitudes towards health risks associated with wild meat. In this case, attitude included perceived beliefs that actors had towards the health risks associated with wild meat and how this influenced their actions.

Data codes were then extracted and placed under the respective data category. By reading through the codes; recurring patterns, ideas, or concepts that emerged from the data were inductively identified, defined, and summarized in Microsoft Excel as themes (S4). We used the emerging themes to conduct a descriptive analysis for generating tables and graphs, and to summarize all the themes emerging from each data category. Additionally, we used the approach by Murungi et al. (2021) [54] to recreate the wild meat value chain by plotting actors, and their interactions into a flow diagram. We demonstrated wild meat flow between actors using arrows, with the arrowhead pointing at the product recipient. Based on a previous study [16], we only report numbers for actors’ health risk awareness levels in the results section to demonstrate within sample trends. However, these should be cautiously interpreted as the use of numbers to report qualitative surveys could be misleading [50]. Therefore, for other variables, any measures of proportions within the sample population are only provided as supplementary information.

The literature search yielded 1093 articles and 16 were retained after removing duplicates and screening against the exclusion and inclusion criteria (Fig 2). Out of the 16 studies, two were thesis reports, two were presentation abstracts while the rest (12) were peer-reviewed scientific articles. All the retained studies were conducted within the NMA and its surroundings. The lead author extracted data on food hygiene and safety practices, knowledge, and attitudes into a Microsoft Excel sheet (S5). We used thematic analysis, as was with the data from key informant interviews, to summarize our systemic literature review findings.

## Results and discussion

In this section, we present our key informant data on the value chain structure and governance, the actors, and their motivations as well as the temporal and spatial dynamics of the chain. We also identify the wild animal species that are targeted along the studied value chain. Thereafter, we present a summary of value chain actors’ practices, knowledge, and perceptions regarding health risks associated with wild meat. Initially, we detail these observations based on responses from actors in the local market. Subsequently, we present our findings on the distant trader node based on the information provided by poachers supplying the distant market, other actors, and lastly from the systematic literature review (SLR).

**Fig 3:**
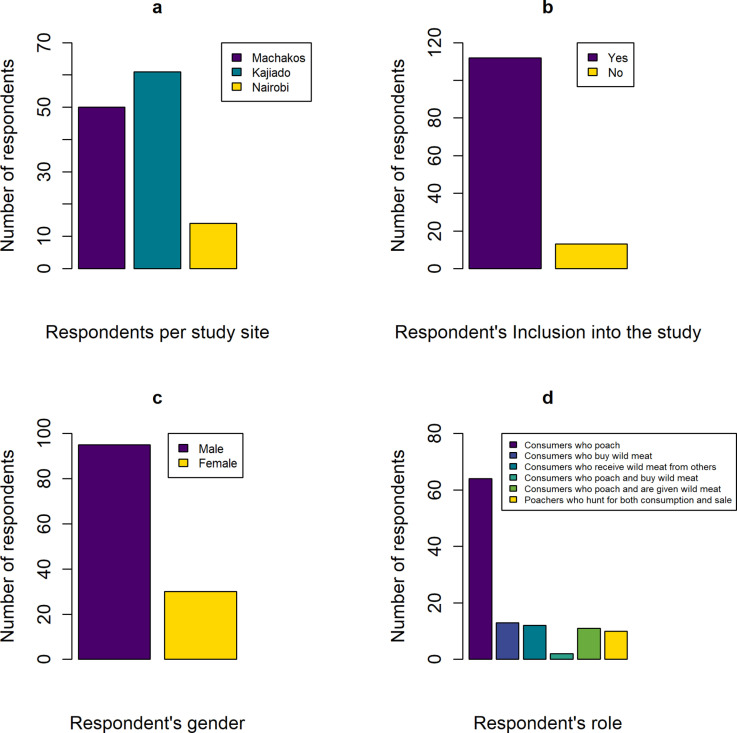
Sample characterization showing (a) Different actors interviewed, their roles and how they interact with each other; (b) Gender of the respondents; (c) Number of respondents included in the study; and (d) Number of respondents per study site.

We approached 125 key informants for interviews. Of these, 112 participated fully in the study while 13 did not complete the interviews, for two reasons. Firstly, respondents (n = 11) requested to be exempted after the start of the interviews for various reasons including lack of time, or due to fear of possible legal repercussions from the interviews. Secondly, the lead author intentionally excluded respondents (n = 2) who showed visible signs of discomfort when responding to the questions. We present the study’s sample characteristics in Fig 3. Out of the 125 key informants approached for interviews, 95 were males and 30 were females. All respondents who completed the interviews (n = 112) had consumed wild meat before. The poachers (n = 97) hunted for consumption (n = 87) as well as for sale locally and to distant markets (n = 10).

### Overview of the wild meat value chain supplying the NMA

**Fig 4:**
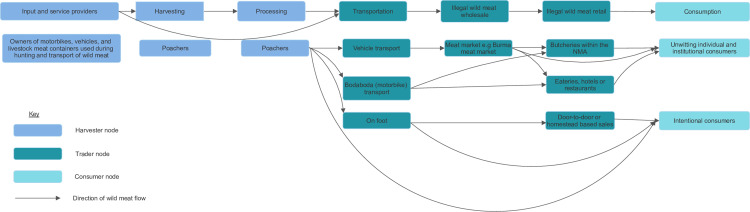
The structure of the wild meat value chain of the NMA showing the different nodes and actors involved.

The general outline of the value chain structure, product flow and actors involved is presented in Fig 4. The wild meat value chain comprised three main nodes: The harvester node dominated by poachers; a trader node comprising meat wholesalers, retailers, seldom transporters; and the consumer node. We had this structure a priori and no data was forthcoming to indicate that it was different. Actors were motivated to participate along the value chain for food and income, as well as for perceived medical and nutritional benefits from wild meat (S1 Fig).

PM010305K-k (Poacher and consumer): *“We do consume but not often, because the meat is rare, but when there’s disease outbreak, like right now when it is raining and people or children have flu, and even during the corona pandemic people intend to go in search of zebra, its oil, to cure pneumonia. We give it to the kids. We believe it is medicinal.”*

However, actors along the studied value chain had overlapping roles and commonly acted across more than one node. The nature in which actors’ roles overlapped is presumably due to the illegal context in which the wild meat value chain in the NMA operates[5] (Kenya Wildlife Act, 2012). The legal restrictions discourage participation in the value chain leading actors to assume more than one role, in more than one node. Indeed, a direct link from harvester to trader, or to consumer node was commonly reported. This, however, is different from the redundant value chains in other countries where limited barriers aided by obsolete laws, unsuccessful or poor implementation of conservation laws [7,55,56] favor participation and creation of distinctive roles along the value chain [9,10,16,26,57]. While conservation to reduce poaching is yet to be fully addressed in Kenya [58], Kenyan laws, prohibiting wild meat harvest, consumption, and sale appears to limit actor participation, potentially mitigating the risk of infectious diseases emerging along the value chain. This assumption must however be interpreted with caution as its applicability in mitigating illegal wild meat trade may be less successful than thought. Creating stringent laws to mitigate illegal activities such as drug use has previously proved less successful than anticipated [59].

**Fig 5:**
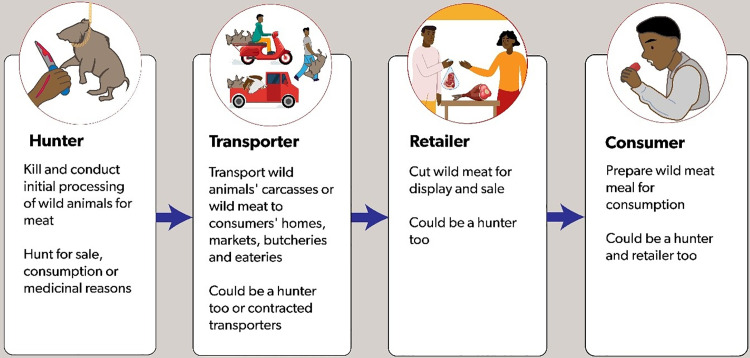
Actors’ interactions with wild animals and wild meat along the value chain. Reprinted from Canva Software under a CC BY license, with permission from Annabel Slater, original copyright (2024).

The reported overlapping roles show that all actors could be equally predisposed to health risks present at any node. This underpins the need for a more general rather than the actor-centered health risk interventions previously recommended in the Democratic Republic of Congo for actors with distinctive roles [16]. Regardless, risk factors for exposure to zoonotic pathogens were higher at the harvester node where poachers increasingly interacted with live or dead wild animals. Poachers hunted, killed, and processed them for meat, sometimes transporting such meat for sale or consumption [8] (Fig 5).

PM020905K-m (Poacher supplying the commercial Nairobi VC and consumer): *“We use torch lights on a motorbike and hooting from the motorbike to confuse the animal and then cut its legs. Once the animal has fallen, we then cut the neck, skin it, cut into pieces and pack in a sack for sale……… Just on the spot. Once the animal has been skinned, the hide is spread on the ground and that is where we place it. After cutting the meat into pieces, we also place them on twigs …….are transported on motorbikes. We take them to a specific person who supplies them, and we just wait for our pay.”*

On the other hand, wholesalers, distant market retailers, and consumers (if not also poachers) encountered wild meat that had undergone initial processing stages, supporting previous findings at the trader node by van Vliet et al. [8]. They would only cut the meat for display, sale, or meal preparation (Fig 5). The longer that meat is handled postmortem the lower the chance of exposure to zoonotic pathogens. However, the risk for contaminant foodborne pathogens [60] from unhygienic handling could increase.

PM021204M (Poacher): *“.... At Burma, the sellers there receive the meat and keep it in coolers, only cutting steak for display, that they sell to customers unknowingly or mixed with other meat…………This is quick cash, especially for the poachers. For the people supplied in Burma, this gives extra profit as wild meat is cheaper than other meats yet when they sell to customers, they charge the cost of domestic animal meat thus more profit.”*

Communities at the study sites attacked, killed, and harvested meat from wild animals that strayed into the community, or invaded farms for consumption. In most cases, such animals were dismembered as everyone cuts a portion of its meat.

PM081204M (Poacher obtaining meat from community kill for consumption) *“I just came across an animal that had been killed and people were cutting its meat, so, I also cut a portion for myself.”*

Additionally, wild meat was obtained from rail and roadkill, from electrocuted animals, remains of predator kill and from animals that died from unknown causes.

PM020805K-Kn (Poacher and consumer): *“Mostly I get wild meat from what remained from hyena hunt……. I only take the portion that has no hyena bite marks because I do not know if the hyena was sick or which germs it has and maybe it could have contaminated the areas it bit.”*

The wild meat value chain in the NMA operated illegally, prompting two types of governance: formal and informal. To control the outlawed practice, the KWS put in efforts towards surveillance of poaching, arrests, and prosecutions of perpetrators. Actors were aware of the KWS presence and the associated penalties including arrests and serving a jail term, or compulsory community service. Two respondents assumed being forced to consume wild meat, raw or cooked, or being beaten as penalties. Most actors considered the formal laws unfair. They reported that wild animals invaded their farms and attacked them and their animals, but they were never compensated, or the compensation was delayed. Consequently, they killed animals for revenge and harvested their meat.

BAG04080523K-KN (Poacher and consumer): *“I am aware that consumption of wildlife meat is illegal as it is enforced by KWS scouts and some of the elders of the area. I have a challenge following such a law because in drought times these animals come here mostly when we don’t have adequate food, so we are tempted to despite the law being clear about consumption. Additionally, these animals staying around here cause destruction such as when elephants destroy property including taps and water pumps at the waterpoints. Furthermore, these elephants also inhibit people’s movement by invading the area and prevent them from carrying out their day-to-day activities.”*

Since actors were aware of the formal laws, they devised informal rules to avoid sanctions. This included sale to only trusted clients, recruiting informants to spy on KWS, operating during hours that limit chances of arrests. Also, they chose transport methods that limited their exposure to the authorities like the use of designated livestock meat containers during transport to disguise wild meat as other meats.

PM031104M (Poacher and consumer): *“And these are people who would not wish to be known and they operate based on trust amongst themselves…………. They organize themselves. They have spies who inform them about where rangers are. As such, they can work quickly and leave before the rangers arrive.”*

Betrayals, like informing the authorities could attract severe punishments from other actors involved, including attacks on the family of the informant, or even death.

PM031204M (Poacher and consumer): *“If you leave that group and you want to tell on them, they [*other actors*] can easily kill you.”*

In contrast to earlier findings that informal governance along wild meat value chains is targeted at profit maximization [7,26], the respondents’ personal accounts of events show that the informal governance in the NMA is aimed at evading arrests. The informal rules that we report supports strategies discussed by Gore et al. [61] in sanction avoidance in the illegal wild meat trade. Regardless, the observed formal and informal governance along this value chain is still largely unfocused on mitigating potential health risks from wild meat. This raises concerns, both at local and global scales, over the risk of infectious pathogen spillover from an illegal urban wild meat value chain operating within a globally well-connected city region [62].

#### Spatial characteristics of the NMA’s wild meat value chain.

Wild meat was harvested from the peri-urban areas within NMA, consumed or sold locally within homes at the poaching hotspots (local market value chain) or supplied to established premises within the peri-urban towns, market centers and to Nairobi City County (distant market value chain) (Fig 1). The demonstrated spatial features indicate potential routes for spread of infectious pathogens from and within poaching hotspots, and along the value chain to other areas within the NMA [63]. Pathogens could also be spread by the value chain actors’ movement as human hosts [64]. The outlined geographical flow of wild meat in the NMA can be leveraged to design spatially enabled pathogen surveillance systems for successful outbreak preparedness and response.

#### Temporal characteristics of the value chain.

Poaching, sale, and consumption of wild meat differed based on seasons. Local harvesting, sales and consumption increased during the dry seasons when animals entered communities in search of water and food. Wild animals were reported to get trapped and killed for meat at irrigated farms during the dry seasons. This was further escalated by scarce food for humans during such periods.

PM040805K-kn (Poacher and consumer): *“Even right now, it is still like there is drought here and everything is hungry. Wild animals are now closer to human settlements because of the available grass and also water and that is how people kill and eat them because we are hungry.”*

Poaching and supply of wild meat to the distant market and locally were reported to increase during periods of higher demand, commonly during Christmas. Due to price inflation for livestock meat during such seasons [53,65]), local consumers resort to cheaply sold wild meat. Unscrupulous traders in the distant market were also reported to source for cheap wild meat that they sell for the price of livestock meat for higher profit. Making the cost of alternative animal protein sources favorable during such times could alleviate dependence on wild meat and mitigate potential spillover [19].

PM061005K-m (Poacher and consumer): *“I consume wild meat may be once and sometime none in a month as I do not hunt unless those animals with arrow or spear wounds from poachers show up in my area …………. During Christmas, the practice is very rampant. I operate a butchery here at Mashuruu. It is really a loss for us during Christmas as wild meat is sold cheaply. Most of it is however sold in those sides of Makindu and Sultan. Us Maasai’s we will always know if someone is selling us wild meat.”*

The enhanced participation in the value chain during the dry season and during Christmas season shows elevated risk factors to pathogen spillover and outbreak during such times. Provision of alternative sources of income and food during seasons of increased value chain activities have been recommended to alleviate the impact of wild meat utilization on biodiversity in Ghana [27]. The recommendations in Ghana [27] could be replicated to mitigate the risk of infectious diseases outbreaks along urban wild meat value chains during such seasons. These could include crime-alleviating short-term youth employment programs such as the *kazi kwa vijana* [jobs for the youth] to boost household income during such times [66]. Continued efforts to close the continued yield gap across crop and livestock production in sub-Saharan Africa are needed [67] and alternative protein sources, such as edible insects [68] and wild animal farming [69] could also be considered. Importantly, behavior change campaigns aimed at reducing reliance on wild meat [29] should be emphasized.

During the early and late growing seasons, wild meat was harvested from animals who invaded farms and destroyed crops, as an act of revenge. In contrast to findings from Ghana [27], that a decline in wild meat harvesting during peak farm activities, our study reveals the opposite trend. This disparity may stem from increased human-wildlife conflicts during growing seasons in the NMA [70] (Wandaka et al. 2019). Wild animals encroach on farms, leading to conflicts with communities, who subsequently kill and harvest their meat. Initiatives that would limit cases of human-wildlife conflict are therefore key. Initiatives could include targeted fencing off of wildlife (Nyongesa et al. 2008) [71] as well as use of crop-protection strategies and tools as was demonstrated in the human-wild animal interphase in Kilimanjaro, Tanzania.[72] (2019).

PM131804M (Local poacher and consumer) *“For the quail or the yellow-necked Francolin [Francolinus leucoscepus] and the guinea fowl [Numinidae], they are mostly hunted during the growing season as they eat the planted seeds……. Mostly the hare, and zebras too are invading the farms during that time.”*

#### Animal species targeted for wild meat and species deception.

A diverse assemblage of wild animals, ranging from farm rodents and pests like hare, squirrels, porcupines, birds and medium to large size mammals were targeted (S2 Fig). Birds in the family *Numididae* (guineafowl), *Columbidae* (doves) and *Anitadae* (ducks), *Phasianidae* (francolins and quail) and *Struthionidae* (ostrich) were hunted for meat. Ungulates, particularly *Phacochoerus africanus* (warthog), *Gazella spp.* (Gazelle)*, Aepyceros melampus* (impala), *Tragelaphus imberbis* (lesser kudu), *Connochaetes taurinus* (wildebeest), *Alcelaphus buselaphus* (hartebeest), *Syncerus caffer* (buffalo), *Taurotragus oryx* (eland), *Equus quagga* (zebra) and *Giraffa camelopardalis* (giraffe), and rarely *Loxodonta africana* (elephants) were also target species. The *Panthera leo* (lion) was the only carnivore species hunted for meat, and for its fat which was perceived as medicinal by the local communities.

For local consumption, respondents in Kajiado reported less preference for smaller sized animals like *Lepus timidus* (hare), most birds (except *S. camelus)*, likening them to *Gallus gallus domesticus* (chicken)) and fish. Chicken and fish are culturally less preferred meals by the Maasais.

PM030805K-kn (Poacher and consumer): *“Just culturally, we believe that hare is meat for the cat and the dogs.”*

Meat supplied to the distant markets was however preferably harvested from large animals like *G. camelopardalis*, *E. quagga*, *S. caffer*, *T. oryx*, and *A. buselaphus* (S2b and 2c Fig). This can be explained in two ways. First, poachers supplying distant markets indicated that traders preferred wild meat that would easily pass as meat from livestock. This was corroborated by some consumers who reported to us they would avoid some butcheries in the distant markets as they suspected they could be covertly selling wild meat. In the distant markets therefore, value chain actors were thus denied the chance to make informed decisions and take precautions [73] further increasing their risk factors for exposure to pathogens from wild meat. This practice is called “species deception” [73] and is an act of food fraud [74] targeting unwitting customers in the NMA. We hypothesize two differing drivers for species deception. First, by selling wilds meat disguised as other meats, such sellers could easily stay hidden from the authorities and avoid arrest. Secondly, the cost of obtaining wild meat may be relatively cheaper as it does not involve the financial costs incurred in raising livestock [75]. This therefore makes illegal wild meat more profitable, if they are sold at the market price of a more expensive domestic animal meat. Such substitution food fraud can be found globally, with the 2013 EU ‘horsemeat scandal’ being a high-profile example of such [76]. Creating public awareness about food fraud and the importance of buying meat from only reliable sources could potentially alleviate the health risk faced by the unwitting customers [77,78].

PM032104M (Poacher and consumer): *“I was coming to that. So, when there is a roadkill here, especially as it was during the last drought season, motorbike riders will flock the scene, with sacks and the lorry drivers and in most cases, the wild meat is eaten in these local centers by unknowing customers………... Yes, here in Malili, in Makutano junction and Athi River. The meat is sold backstreets to meat vendors and the hotels and to homes. Mostly, when you go to hotels in these areas, when you order meat, you find that the wild meat like giraffe or wildebeest or gazelles’ meat is mixed with either goat or beef and served to you and you just eat unknowingly.”*

The large ungulates like *E. quagga*, *G. camelopardalis* and *T. oryx* yield more meat per poaching operation. Harvesting from such large ungulates was therefore preferable over small size-ungulates as it reduces the need for frequent hunting, as a measure of sanction avoidance [61].

PM020905K-m (Poacher): *“Because zebra and giraffe are big and have more meat, it limits the need to hunt more often. Also, one can easily pass check points by saying that they are transporting beef since the pieces looks like beef.”*

Unlike traders in the distant market, local retailers were known by consumers to be selling wild meat. In some cases, they would inform buyers about the species from which they obtained meat. Some buyers would also ask about the species from which wild meat was obtained. However, they were not entirely sure if they were told the truth. A few local consumers had the knowledge of how to identify wild meat, and the species it was from. Such consumers only bought wild meat knowingly, and would avoid retailers, butcheries, eateries, or outlets in the distant markets that they suspected of selling wild meat covertly. Regardless, the target species that we report for both the local and distant markets mirror previous reports in [21,40]. Hence, it could conceivably be hypothesized that the main potential source of infectious pathogen spillover and outbreaks in the NMA is ungulates. Pathogen surveillance efforts should target these groups of animals for timely mitigation.

### Overview of practices and health risk perception along the local wild meat value chain

#### Actors’ poor practices with regards to the health risks of wild meat.

Respondents participating in the local harvest, consumption and trade of wild meat reported several practices that could be considered as health risks. None of the actors interviewed wore PPE while handling wild animals or wild meat. Most respondents, especially poachers, reported not washing their hands either before or after handling wild meat. Wild animals were also slaughtered on the bare ground. During transport, wild meat was reportedly carried on the bare back, around bare necks, hooked on a stick, in sacks, or lined around the body then covered with clothes to avoid drawing attention. Some poachers would package meat from different species together when coming from hunting trips.

PM081104M (Poacher and consumer): *“Yes, meat, from any wild or domestic animals are all meats, so at times, we could just mix meat from different wild animals and prepare them together as one meal or mix even with meat from domestic animals.”*

Poor food safety practices by local retailers were reiterated by the consumers who bought wild meat from them. They noted that the local retailers had poor hand hygiene status, sometimes they wore dirty clothes and packaged wild meat in dirty or blood-stained sacks and in plastic jerricans. They also did not wear any PPEs during the sale.

PM022604N (Poacher and consumer): *“He was carrying the meat in a sack. It was just a stranger that came by and asked me to buy meat that they are selling, but even from personal appearance, I would not say they were hygienic. Just an ordinary person and you just cannot tell their way of life.”*

Consistent with previously reported attitudes [16], most respondents thought it was not necessary to adhere to safe practices because that was not how they were used to doing things. In most cases, they reported lack of time to consider any safety measures as actors feared being arrested by KWS.

PM071204M (Poacher and consumer): *“We never washed our hands; we were rushing to leave the scene to avoid arrests and to ensure we get a piece before it is all gone. So, there was no time to consider any of that.”*

Additionally, formal meat inspection was not conducted due to the illegal nature of the value chain. Interestingly, a few respondents mentioned measures that they perceived as food safety checks for wild meat (Table 1). These included visual inspection for body condition, presence of cysts or worms in the viscera, muscles, or gut, enlarged liver, pancreas, presence of blood, changes in color of meat or visceral organs.

**Table 1:** Safety checks for health risks in wild meat traded in and consumed along the wild meat value chain supplying the Nairobi Metropolitan Area as reported by value chain actors.

Method of safety checks	What to look for	Indicators of safety status of wild meat	Decision
Visual check	Changes in color	Green color in meat or viscera	Not safe for consumption
		Brown coloration of pancreas	Meat not safe for consumption
		Red coloration of meat	Fresh meat thus safe for consumption
	Worms in the gut or muscles	Presence of worms in muscles	Not safe for consumption thus throw meat away
		Presence of worms in muscles	Not safe for consumption thus thoroughly boil the meat before consumption
		Presence of worms in muscles	Not safe for consumption but just consume anyway
	Appearance of organs PM080805K-Kn (Poacher and consumer): “*When it (the pancreas) is enlarged, then the meat is not okay. They also blow air into the pancreas and if it turns brownish, then it is okay, but not okay for any other color.*”	Enlarged liver, pancreas	Not safe for consumption thus the organ, but not the meat is disposed
		Fluid filled lungs	Not safe for consumption thus the organ, but not the meat is disposed
	Animal’s body/carcass appearance	Thin animals	Avoid whole animal or just take specific parts like the hind limbs
		Presence of predator or dog bite marks	Avoid bite mark areas only
Touching wild meat	Consistency and texture of meat	Wet feeling on meat	Wild meat is fresh thus safe to consume
		Dry feel on meat	Wild meat may not be fresh
Smelling	Changes in smell	Off odors	Meat is stale
			Meat is from wild animals and not domestic animals
Burying meat under sand Observing meat during boiling	Meat popping out from under the sand Dropping out of cooking pot PM091204M (Poacher and consumer): “*You cannot hang hare’s meat out in the sun as it may make it have* “ndulu [Kamba name for anthrax]” …………. *It is a condition in which when you start cooking the meat, it keeps on ‘jumping out’ of the sufuria…… If you eat such meat, you get sick*”.	Meat popping out from under the sand or ‘jumping’ out of cooking vessel	Not safe for consumption (assumed to have Anthrax); disposed
Eating the wild meat or feeding to cat or dogs	Health outcome of the person or animal who consumed the meat	If the person, the cats, or dogs are okay	Meat assumed to be safe
Assumed inspection by someone in authority	Permission to consume wild meat. PM051904M (Consumer): “*If the person in charge gives us a go ahead to harvest meat from the wild animal carcasses, we believe they have inspected it, and it is safe*.”	Permission to consume wild meat is given	Meat is safe

The poor wild meat handling practices we report have also been documented in other African countries [8,25,28]. Handling wild meat without PPE puts actors in direct physical contact with animals and their tissues ([22]. Consequently, they could get exposed to pathogens in wild meat, leading to pathogen spillover from the wild animals as was hypothesized for SARS-COV2 [79]. In addition, the demonstrated poor personal and utensil hygiene could lead to wild meat contamination [22] and subsequent foodborne illnesses.

Some consumers perceived roasting, sauteing and boiling for shorter durations as ineffective methods of cooking wild meat supporting findings from other studies [80,81]. They reported that such cooking methods could predispose one to infections from retained tissue fluids in the undercooked inner parts, or toxins in meat. However, roasting was still considered for specific animals such as birds, young or small sized mammals like hares and squirrels.

PM030805K-kn (Poacher and consumer): *“So, after eating swara* [term used by the locals to refer to antelopes] *meat from a carcass, they got sick. Later, we learned that the swara had died ………… they had diarrhea and stomachache, they were like 7 guys. When they went to the hospital, they were told the swara had snake poison in it and that is what made them sick, possibly because they did not boil it to get rid of the poison. One of them died and that made people fear eating meat from wild animal ………. We believe that boiling helps and what made my friends sick is because they did not boil it. They were herding and they did not have a sufuria* [saucepan] *so they just made fire and roasted it, yet it had the snake poison.”*

Boiling was mostly preferred to make meat tender, to rid it of toxins, as a family tradition, and seldom to eliminate germs. Respondents who boiled wild meat discarded the broth after boiling as a typical family cooking method, because it was not tasty, and sometimes as an effort to rid wild meat of toxins or germs. However, none of the respondents reported any standard way of knowing when meat is properly cooked.

PM032104M (Poacher and consumer): *“When you boil the meat, all the blood, other body fluids and germs go into the broth. By discarding the water, those are also discarded. It is just like the bitter vegetables; they must boil first and all the broth from the first boiling discarded to reduce the bitterness.”*

A few respondents, specifically at harvester node mentioned eating raw meat (kidney, liver) as an oath taking ritual amongst hunting group. When hunting and hungry because there is no fire to cook the meat with, these visceral organs were eaten raw. This indicates a higher risk of exposure to pathogens from wild meat at the harvester node [24].

PM111804M (Poacher, retailer, and consumer): *“Yes, so during hunting, there are some parts that we ate raw …… The kidney, liver…... Because when you participate in that, it is like a ritual, we believe that after that, you cannot tell on or betray the group.”*

At home, traditional short- and long-term food storage methods were used. Short term storage methods included boiling meat with or without salt until dry and then storing it in a dry place; storing wild meat under a shade; inside a bucket of cold water, or in an enclosed place like the cupboard or in covered containers.

PM101204M (Poacher and consumer): *“You can dip it in a bucket full of cold water or spray it with salt for it to dry……. Yes, i*t [a bucket of cold water] *is like ice.”*

Sun drying, salting, smoking, grilling or a combination of any of these approaches were used for long term storage of wild meat. Preservation of wild meat was aimed at enhancing its shelf life. The storage methods we report have also been reported in wild meat value chains in other countries [7,8,26]. Only two consumers in the local value chain had refrigerators, highlighting likelihood for the persistence and presence of foodborne pathogens at the consumer node due to lack of a cold chain [82,83].

The reported actors’ poor food safety practices during harvesting, processing, transport, meal preparation and consumption are presumably due to actor’s limited knowledge levels [25,84] as discussed in the subsequent section. However, poor practices could also have been partly motivated by sanction avoidance. Actors would ignore safe meat handling practices to reduce the time they spent doing the prohibited practice and consequently limit chances of apprehension [61]. The observed interplay between knowledge levels, sanction avoidance and poor practices further underpins the need for the Kenyan government to reassess the implication of conservation policies on the risks of disease emergence from covert harvesting, consumption, and sale of wild meat [24,85].

#### Limited knowledge and perceptions of the health risks from wild meat.

Here, we report findings from all respondents, including poachers who supplied the distant markets. A majority (76/112) of the study participants who completed the interviews were aware that they could be exposed to health risks when they interact with wild animals and meat. However, they were less aware of the specific risks they faced, complementing findings in Indonesia [86]. For instance, only a few of them could associate health risks with clinical signs (46/112), specific diseases or pathogens (25/112), injuries (26/112) or risk factors to infections (16/112) attributable to wild meat (S3 Fig).

The observed limited knowledge about the specific health risks from wild meat that we report here could be explained in two ways. First, in Uganda it was reported that, actors were more aware of the specific health risks from wild meat due to the occurrence of wildlife related epidemics in Uganda [25]. In Kenya, however, no epidemic had been associated with wild meat or wild animals before, at least not originating from Kenya. The absence of specific outbreaks associated with wild meat potentially explains the limited knowledge levels [22] (. Creating awareness on the specific health risks of wild meat amongst actors and participating communities is therefore key. Secondly, consumptive wild animal use in Kenya is illegal. Any disease incidents from wild meat are therefore unreported. Victims avoid seeking treatment or they lie about the actual cause of illness out of fear of arrest. Failure to report infectious disease outbreak incidences impedes disease surveillance and documentation efforts [87,88]. These further limit the ability of the country and the public to recognize the specific health risks that lies in the interactions with wild animals and their products. Finding feasible trade-offs between implementing laws against illegal consumptive use of wild animals and the need to mitigate the risk of infections could encourage disease incidence reporting [2].

Knowledge of health risks seldom included specific diseases such as Anthrax, Rift Valley Fever, Amoebiasis, Typhoid, worm infestation, diabetes or cysticercosis, but was majorly about the signs of illness. The clinical signs reported in this study included stomachaches, vomiting, diarrhea (could be bloody), constipation, joint, back and body pain and weakness, fever, mental disorientation, skin rashes, pimples, and perceived allergies.

PM012104M (Poacher and consumer): *“I once had this employee who ate wild meat and fell ill… He got affected in the whole body, fever, it was severe, but I could not tell which disease it was…… It really affected him. He stopped working for me and eating wild meat. I could have also been infected, but he had eaten the whole liver.”*

The signs of gastrointestinal disturbances frequently reported in this study could be indicative of foodborne pathogens [89]. Foodborne pathogens, capable of gastrointestinal disturbances, have been extensively documented as wild meat contaminants [4]. The risk factors of exposure to foodborne pathogen infections to consumers was evident in this study considering the unhygienic food handling practices actors engaged in. This further underpins the need for actor education on safer and hygienic practices during handling of wild meat.

Surprisingly, actors attributed all signs of illness and the few specific reported diseases or pathogens to wild meat consumption, but not handling. However, injuries were all attributed to handling of wild meat, adding to the existing evidence on the risks of occupational hazards along wild meat value chains [22,25]. Similar to consistent with findings from a previous study [16], actors were less worried about injuries as a health risk and thus downplayed it as a means of exposure to pathogens from wild meat

PM081204M (Poacher and consumer): *“So many wildebeest were attacked and killed here in the community just in last dry season alone. For example, on the way to Mitaboni, I hear that around 40 wildebeest were killed in just a day…. There were lots of injuries. Some people’s hands were chopped off as people struggled and scrambled for a piece of meat.”*

### Practices and health risk perceptions at the distant market

#### Distant market’s harvester node.

Poachers supplying the distant market interacted with wild meat in similar ways as they did with meat intended for local sale or consumption. They trapped, killed, slaughtered and eviscerated wild animals before cutting the meat into pieces and packaging these for transport. Some poachers also acted as transporters while wholesalers and retailers at the trader node could also contract transport services.

PM021204M (Poacher supplying the distant market, and a consumer): *“You can use motorbikes. At first, we used to use motorbikes, just tying the meat on the bike after hunting, and transporting it. However, things changed later, and we started using probox [*a vehicle*], pick-up trucks especially to transport meat to Burma. At Burma, the sellers there receive the meat and keep it in coolers, only cutting steak for display, that they sell to customers unknowingly or mixed with other meat.”*

Just like in local sales, poachers supplying the distant markets reported using unclean hands and utensils to handle wild meat. They slaughtered on the bare ground, at night and did not wear PPE. They packaged wild meat in sacks, nylon bags, in designated livestock meat containers for transport via motorbikes or cars, or sometimes directly on bare car trunks. The choice of how meat was packaged was driven by the need for sanction avoidance. The use of waterproof bags, though rarely for food hygiene, helped in evading arrests as blood-stained bags would draw attention. Similarly, use of the standard metallic meat boxes from livestock slaughterhouse helped disguise wild meat as other meat.

Poachers supplying the distant markets did not have or seek any official meat inspection, with only one reporting minor safety checks. These poachers attributed paucity in compliance to safe food handling to hunting at night, mostly in a rush to avoid arrest. They also just did not care about it.

PM070505K-o (Poacher supplying the distant market, and consumer): *“When supplying to other areas after hunting, the hygiene and safety of the meat during transport is not my business. I only hunt. Meat is always picked up by vehicles fitted with red and white colored meat boxes for transporting farm animal meat from slaughterhouses, or just sacks. That is what they are transported in very early in the morning, especially to Burma.”*

As outlined earlier, poachers supplying the distant trader node also had limited knowledge about the health risks from wild meat. This, in addition to lack of an enabling environment for safer practices, as prompted by the need for sanction avoidance, explains their reported poor practices.

As understood by some of the respondents, distant market retailers operated as meat vendors in butcheries, hotels, and eateries including the *nyama choma [*charcoal grilled meat*]* joints. They interacted with wild meat during storage, display and sale. They purchased wild meat intentionally from poachers but sold it disguised as meat from domestic animals. Wholesalers, reported to be mainly at the Burma market, were also supplied by poachers and they covertly sold wild meat to other retailers, hotel or eateries owners, street food vendors and to consumers. This finding supports our preliminary data, and previous studies [40,42] that wild meat in some towns and market areas in Kenya is sold disguised as or mixed with livestock meat.

PM031204M (Poacher supplying the distant market, and consumer): *“……I do not know but I guess may just how they handle other meats in the shop. You know, this is an illegal practice, and you cannot put a notice on the shop that you are selling wild meat. So, they are sold disguised as other meat to avoid attention”*

#### Distant market’s trader node: A review.

In this section, we report our findings from the SLR to give potential insights on health risk practices, knowledge, and perception for vendors at a node where wild meat is covertly and fraudulently sold. Amongst the 16 studies reviewed, 12 concluded that there exist poor practices amongst meat vendors in the NMA, further recommending the need for training on hygienic and safe food handling practices (S5).

Four studies conducted on informal premises reported lack of adherence to PPE [90–93]. Lack of adherence to handwashing was reported by five studies in similar premises [91–95]. These practices were also associated with increased food contamination [94,96]. On the contrary, two studies, conducted in the formal premises reported provision and adherence to PPE and handwashing by food handlers [97,98]. For informal meat vendors who could also be selling wild meat, lack of PPE would put them into direct contact with tissue and fluids from wild meat. Lack of handwashing was shown to cause cross-contaminations from hands, as well as vendors’ infections from unintentional ingestion of contaminant pathogens [96,99,100]. Educating food, including meat vendors, especially in the informal markets, on the importance of hand hygiene and PPE could alleviate potential spillovers from wild meat that are sold fraudulently and concurrently with or disguised as livestock meat [96].

Cross-contaminations could also stem from poor premises, utensil, and surface hygiene at any meat vending site [100]. Higher prevalence of poor utensil, equipment and surface hygiene was reported by ten of the studies reviewed (S5), with only three studies on formal premises reporting the opposite [91,97,101]. These included the use of dirty chopping boards, knives, poorly cleaned surfaces, meat transport boxes, among others. Poor utensil hygiene was highlighted as a food safety risk at the poultry trader node by [102]. Poor personal hygiene practices, including having long nails, reuse of oil and water to cook or clean different types of foods, blowing of air into polythene packaging bags using mouth and con-current money or phone and food and meat handling were also reported amongst the vendors.

Most vendors operating informal premises were also reported to lack proper food storage measures during transport, vending and after sales (S5). Different meat types, including offal, could be stored together [103], with concerns being raised over the use of meat transport boxes to carry food items other than meat [93]. From the key informant data, it was reported that wild meat suppliers used designated livestock meat boxes to transport wild meat. This raise concerns over cross-contamination from wild meat to the other meats or any other food transported in such containers. Strengthening public health regulations governing the transport and storage of meat could weed out the leasing of livestock meat transport for transporting wild meat. This will require a multisectoral efforts by the KWS, the directorate of veterinary services (DVS), Kenya, and the public health departments in the NMA.

Similarly, strengthening laws and infrastructures to create an enabling environment for safe meat vending could encourage better practices by meat vendors [104]. Studies reviewed reported the presence of dirt, open damping sites, dust, insects, pests and rodents around the vending or food handling area. The lack of toilets, hand washing facilities and clean treated and running water were other contributing factors. In premises where these were provided with and compliance reported, Improved practices and reduced contaminations were reported in premises where an enabling environment for compliance to food safety standards existed [94,97,101]. Additionally, the legal environment had laws that were poorly implemented, consequently leading to poor compliance as was reported with medical certificates (S5).

Amongst studies that assessed and reported on knowledge, perceptions, and attitudes (n = 9), it was noted that education had a positive impact on food safety practices (S5). More educated meat and food vendors or handlers were more likely to have improved food handling practices. Formal meat handling premises hired trained personnel, most of whom had knowledge of potential health risks, including diseases associated with unsafe food handling practices. This therefore underpins the role of awareness creation in mitigating zoonotic risks from meat. Interestingly, some believed that it is their duty to ensure food is safe [91,95,97], while others assumed that they are not responsible so long as customers do not demand for quality [54]. Regardless, vendors at this node, especially those operating informally, still engaged in poor and unsafe practices. This could be attributed to weak policy implementation by the NMA public health department commonly due to corruption as has been documented elsewhere Wild meat is a major source of livelihoods and nutrition in the tropics, [104]. Strengthening policy implementations could potentially discourage the reported poor practices and mitigate potential health impact from illegal wild meat sale at the distant market’s trader node.

## Conclusion and study implications

Our study reports on the structure and features of an illegal wild meat value chain operating in one of Africa’s largest cities; the Nairobi Metropolitan Area. Ungulates were primarily targeted for their meat, which was sold and consumed locally or supplied to distant markets. Actors along the studied value chain displayed paucity in compliance with food hygiene and safety protocols, presumably driven by limited knowledge of the health risks from wild meat and sanction avoidance. They therefore put themselves and the urban residents in the NMA at risk of being exposed to pathogens from wild meat. The nature in which different urban centers are interconnected globally, via land, water, and air travel, makes the wild meat trade and consumption in the NMA not just a Kenyan but a potential global health problem. We recommend creating more awareness amongst value chain actors about the specific health risks of wild meat through public education. Most importantly, the government of Kenya should apply systematic and integrative approaches to concurrently prioritize mitigation of public health risks at the wildlife conservation, food security and health nexus. For instance, collaborations between the communities, personnel in the Ministry of Health and the KWS, amongst other stakeholders, can be very crucial in multisectoral infectious pathogen mitigation efforts. Our recommendations can be leveraged in timely prediction, preparedness, response and recovery from future wild meat related pandemics.

## Supporting information

S1 File Project information sheet and the consent form. (DOCX)

S2 File Guide for key informant interviews used during the study period. (DOCX)

S3 File PRISMA-P checklist for Systemic Literature Review (SLR) on knowledge of, attitude and practices towards meat hygiene and safety by meat handlers in the NMA. (DOC)

S4 File Data templates demonstrating summarized thematic areas that emerged from the key informant interviews. (XLSX)

S5 File Data extraction sheet for Systemic Literature Review (SLR) on knowledge of, attitude and practices towards meat hygiene and safety by meat handlers in the NMA. (XLSX)

S1 Fig Respondents motivation to participation in the wild meat value chain supplying the Nairobi Metropolitan Area (NMA)
(DOCX)

S2 Fig Frequency of wild animal species (a) respondent targeted along the value chain supplying the NMA; (b) that respondents knew were poached and supplied to the distant market; (c) that poachers hunted for wild meat they supplied to distant markets.
(DOCX)

S3 Fig A graphical representation of the awareness levels for the specific diseases/pathogens, clinical signs, injuries, and the risk factors to infections that respondents were aware of as health risks attributable to wild meat.
(DOCX)

S1 Data Registered Review Protocol. (PDF)
