# Supplementary material for: Limited knowledge of health risks along the illegal wild meat value chain in the Nairobi Metropolitan Area (NMA)
Source: PLoS One. 2025 Mar 26;20(3):e0316596. doi: 10.1371/journal.pone.0316596 (PMC11940438; doi:10.1371/journal.pone.0316596)
Supplement: S1 File — (DOCX) [file pone.0316596.s001.docx]

**Participant Information Sheet**

**Study title: Health risks associated with urban wild meat in Nairobi**

You are kindly requested to be a part of this research study. This study is conducted by the International Livestock Research Institute (ILRI) in collaboration with the Kenya Wildlife Services (KWS), Wildlife Research and Training Institute (WRTI), the University of Nairobi and Wageningen University. We advise that you read and understand the information below before volunteering to participate. In case you need assistance, the enumerator or a person of your choice will read and explain the project’s details to you. The enumerator will respond to any question you may have regarding the details given below and leave you with a copy of this document.

**1. What is the purpose of the study?**

The aim of this study is to identify health risk associated with wild meat consumption within the Nairobi Metropolitan area. This will be achieved through looking into the wild meat value chain structure and characteristics, its governance and practices and relating this information to data on food hazard risks that are associated with wild meat. The information from this study will help in identifying critical control points for zoonotic and or foodborne illness outbreaks within the Nairobi Metropolitan area.

**2. What is the role of Kenya Wildlife Services (KWS) in this study?**

KWS, through the WRTI are our partners in this project and their main aim is to understand the health risks and hazards that you as a handler or consumer of wild meat, their officers and the communities living together with wild animals are exposed to when you, the community and the KWS officers interact with these wild animals or wild animal products. This project will also help the KWS understand better diseases that could be existing within the wild animal populations. KWS therefore consider this project as purely research; they will only receive anonymised data and will not be able to identify you or be able to prosecute you based on the information you will share with us during this research project.

**3. Why have I been chosen?**

You have been selected to participate in this study because you are a resident of the Nairobi metropolitan area and have at some point been involved in wild meat handling possibly as a hunter, retailer or consumer of wild meat. You are therefore in a better position to respond to our questions. There are other participants who have also been selected to participate in this study but none of them will ever know the information that will be given by you.

**4. Do I have to take part?**

Your participation in this study should be voluntary. If you accept to be a participant, you will be required to give us an informed consent to participate, verbally or written. If you opt to sign but you are unable to sign, your thumbprint impression from an ink rubberstamp will be used instead. If you are unable to read and or understand the consent form and therefore issue consent, a witness of your choice is allowed to do it on your behalf.

**5. What will happen to me if I take part?**

If you accept our request to participate in this study, I will ask you some questions regarding your participation in wild meat trade and consumption and any other persons involved to understand the operations, and the practices that surrounds these operations. The questions will take less than 30minutes of your time and your response will be captured on an audio recorder or taken down as notes. Your response will be recorded to help the enumerators remember them later but will not be linked to you directly and will be treated as the property of this project. The recordings and the notes will later be written into texts, anonymised, stored in a password protected devise and eventually be summarized to show the overall findings of this study. Thereafter, the notes will be shredded or burned, and the recordings deleted permanently.

**6. What are the possible disadvantages and risks of taking part?**

You may be concerned that your participation in this study might allow law enforcement officers to identify you in the future. We will not collect any personal identifying information without your consent. Any identifying information will be maintained only by the ILRI researcher and will not be shared with other people. The data we collect from you in this study will be kept confidential and anonymised before sharing with persons within or outside the project.

If you consent to be a participant, we will request to take off some time of your schedule and this should not be more than 30 minutes of your time.

**7. What are the possible benefits of taking part?**

There are no direct benefits to participating in this project at a personal level. However, the information we receive from you will enable us to understand better the wild meat value chain, its actors and governance and identify possible pathogens contaminating wild meat. Lastly, we will use the information to assess how food hazard risks (risk of pathogenic infections) vary along the value chain. Eventually, this information will help the government to identify critical control points for zoonotic and or foodborne illness that may emerge from the wild meat value chain in the Nairobi Metropolitan area and this will translate to better health security for the participants of this study and their communities

**8. What will happen if I don’t want to carry on with the study or I do not want to answer specific questions?**

You are free to withdraw from the study if you feel you cannot carry on with it. In addition, you are free to refuse answering questions that you feel uncomfortable answering. Your withdrawal or failure to answer some questions will not attract any penalty whatsoever nor will it have any impact to your role as an actor in the wild meat value chain in the Nairobi Metropolitan area. If you withdraw from the study before completion of the interview, any information that I would have collected from you will be destroyed and or deleted in your presence. However, if you withdraw from participating after completing the interview, I will retain your responses.

**9. Who will pay the costs that I may incur through participating in this study?**

You will not incur any cost by participating in this study. Therefore, there will be no compensation or remuneration for being a participant in this project.

**10. What will happen to the results of the research study?**

Anonymised data will be managed by the Wildlife Ecology and conservation Chair Group, Wageningen University. All data will be granted anonymity i.e., personal data that could identify you, your work or where you stay will not be published or shared. The anonymised results will be published in a thesis, study reports, online and communicated between partners. Summarized data will also be presented to key stakeholders, including you as a participant through trainings or in printed form.

**11. What if something goes wrong?**

In case of any concern, follow-ups or any question regarding this project and also concerning your rights as a research participant, kindly reach out to us using any of the contact information on the contact card that we will leave with you

**Thanks for participating!**

INFORMED CONSENT FORM

| Project Identity: “IREC2022-39”  Principal Investigator: Dr. Annie Cook |  |
| --- | --- |

|  | **Please initial box** |
| --- | --- |
| 1. I am an adult (18 or and above years old) |  |
| 1. I have read (or been read to) and understood the participant information sheet for this study. |  |
| 1. I understand that my participation is on a voluntary basis and I am free to withdraw from participating without my legal rights being compromised. |  |
| 1. I understand that the information that I will share will be kept confidential and I therefore allow you to audio record my response |  |
| 1. I understand that the information that I will share will be kept confidential and I therefore allow you to take notes during the interview |  |
| 1. I understand that the information I am sharing with you will be granted anonymity |  |
| 1. I agree to take part in this study through a verbal consent |  |
| 1. I agree to take part in this study by signing this form. |  |
| 1. I agree to be contacted in the future for any follow-up exercise regarding this project |  |

| Participant’s name |  | Signature/Thumbprint |  | Date |
| --- | --- | --- | --- | --- |
| Name of Person taking consent |  | Signature |  | Date |
