# Supplementary material for: Limited knowledge of health risks along the illegal wild meat value chain in the Nairobi Metropolitan Area (NMA)
Source: PLoS One. 2025 Mar 26;20(3):e0316596. doi: 10.1371/journal.pone.0316596 (PMC11940438; doi:10.1371/journal.pone.0316596)
Supplement: S2 File — (DOCX) [file pone.0316596.s002.docx]

**Guide for wild meat value chain actors’ interviews.**

**Study Title: Health Risks Associated with Urban wild meat in Nairobi, Kenya**

**Questionnaire ID:**

**Enumerator ID:**

**Project’s Field Coordinator in charge:**

**Date:**

**Area:**

No

**Did you get consent from the respondent?**

Yes

**_____________________________________________________________________________________**

*The questions below should only be a guide for you to direct the conversation and ensure you are getting much detail as possible. However, the conversation should be within the project’s objectives. Ensure all questions as regards to the actor role (*hunt/consume/sell*) are responded to, especially for overlapping roles.*

**A. Wild meat VC characteristics**

*In this section, I will ask you questions regarding* ***your role(s) in the wild meat value chain, other actors involved, species targeted, and the temporal and spatial characteristics of the wild meat value chain. I will repeat some of these questions based on any additional role(s) you play along the value chain.***

1. From what species of wild animals is the meat that you have **hunted/consumed/sold** obtained from?

-Why? (*Probe for existing demand, financial, cultural, medicinal reasons, and inherent animal traits (size, taste, et. c) making people prefer the meat)*?

1. Why do you **hunt/consume/sell** wild meat?

-How often do you **hunt/consume/sell** this type of wild meat? Why?

-Are there particular times of year/and day when you **hunt/consume/sell** this meat the most? Why?

1. If you obtain the wild meat you **sell/consume** from others, do you know where they acquire that meat from?

-*Probe; is meat obtained within Nairobi Metropolitan Area and the specific areas or from outside NMA*

-If they respond that they obtain meat from a specific person probe; (*Is that a butcher or a hunter? Where is this person located? Do they operate from a fixed location or are they moving around? Do they sell anything other than wild meat? Why do you buy from them, probe for trust, hygiene, price, loyalty, and established relationships?*

*-*If they obtain it as gift, *ask about who gifts them*

1. If you hunt, are there specific places you hunt from? Why?
2. What status is the meat in when you get it during **hunting/for sale/ for consumption**?

-For poachers, *probe for the status the animal they harvest meat from is in during harvest.*

-*Probe retailers and consumers for any processing already done or if they obtain whole carcasses, or live animals.*

*-Probe every actor on any additional processing they do upon obtaining meat.*

1. ***Regulations***

*In this section, I will ask you questions regarding* ***any means by which control is exercised along the value chain and to understand the person(s) in charge of enacting and enforcing the control measures.***

1. Are you aware of any regulations governing the trade in and consumption of wild meat? (*Probes; what are some of these regulations? Are these regulations enforced? By whom? Do you experience any challenge in complying with the regulations?)*
2. Are there any rules that govern your participation in the wild meat value chain and are not enacted by the government? How are these laws created and by whom?

**C. Actor knowledge, attitude, and practices**

*I will ask you questions regarding* ***your knowledge and attitude towards the health risks that are posed by the wild meat along the value chain. I also hope to learn more about some of the practices you engage in, and if they could be relating to the health risks posed by wild meat.***

1. Are there any hazards you are exposed to as you interact with wild meat?

-*Probe for*

*-during harvesting (****if actor hunts for wild meat****)*

*-during sale of wild meat (****if actor sells wild meat****)*

*-during preparation and consumption of wild meat (****if actor prepare and consumes* wild meat**)?

-If yes for any category, *Probe on hazards* ***due to;***

*-Injury and diseases or pathogens that they know they could contract, or clinical signs they could exhibit from their interactions with wild animals or wild meat*

*- specific diseases, associated pathogens, or signs of illnesses they have ever experienced from their interactions with wild meat*

1. Are there any steps you take to protect yourself and others (including your customers, family, and friends) from these diseases, signs of illness or injuries?

- *For every role an actor is playing, probe on transport, storage and cooking temperatures, storage duration, proximity to other foods during transport, storage or display* *and mixing with meat from other wild animal species conditions? general food hygiene techniques.*

*-Probe if they wear Personal Protective Equipment (PPEs) when handling wild animals or wild meat? Why?*

1. How do you prefer wild meat cooked?

-*Probe how cooking is done at home, and for retailers selling ready to eat wild meat, probe how they prepare wild meat for sale*

-Why is any cooking method preferable?

*-Probe; for grilling, boiling, smoking, fresh carcass consumption, medium done meat, sun drying, et. c.*

*- Ask if there are different preferences for cooking methods for the different species consumed*

1. Do you, or anyone inspects this wild meat before you obtain it?

*-Probe for inspections done during harvesting for poachers*

*-Probe retailers and consumers if they know if inspection is done before they obtain wild meat, or if they do it themselves*

*-Probe for why inspection is done*

1. (**If an actor is selling wild meat**) Do your customers ask specifically for this wild meat during purchase?

-*Probe; do you inform your customers that you are selling them wild meat, why? And the species from which it is obtained.*
