# Supplementary material for: Limited knowledge of health risks along the illegal wild meat value chain in the Nairobi Metropolitan Area (NMA)
Source: PLoS One. 2025 Mar 26;20(3):e0316596. doi: 10.1371/journal.pone.0316596 (PMC11940438; doi:10.1371/journal.pone.0316596)
Supplement: S1 Fig — Respondents motivation to participation in the wild meat value chain supplying the Nairobi Metropolitan Area (NMA) (DOCX) [file pone.0316596.s006.docx]

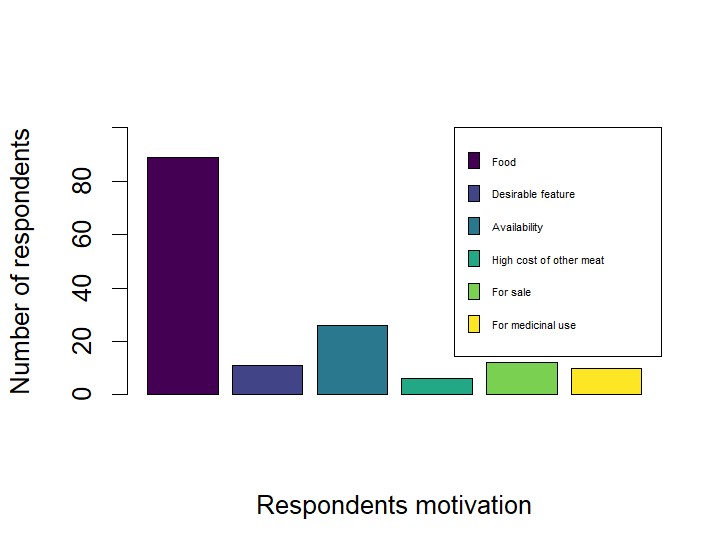


Figure 1: Respondents motivation to participation in the wild meat value chain supplying the Nairobi Metropolitan Area (NMA)
