# Supplementary material for: Limited knowledge of health risks along the illegal wild meat value chain in the Nairobi Metropolitan Area (NMA)
Source: PLoS One. 2025 Mar 26;20(3):e0316596. doi: 10.1371/journal.pone.0316596 (PMC11940438; doi:10.1371/journal.pone.0316596)
Supplement: S2 Fig — Frequency of wild animal species (a) respondent targeted along the value chain supplying the NMA; (b) that respondents knew were poached and supplied to the distant market; (c) that poachers hunted for wild meat they supplied to distant markets. (DOCX) [file pone.0316596.s007.docx]

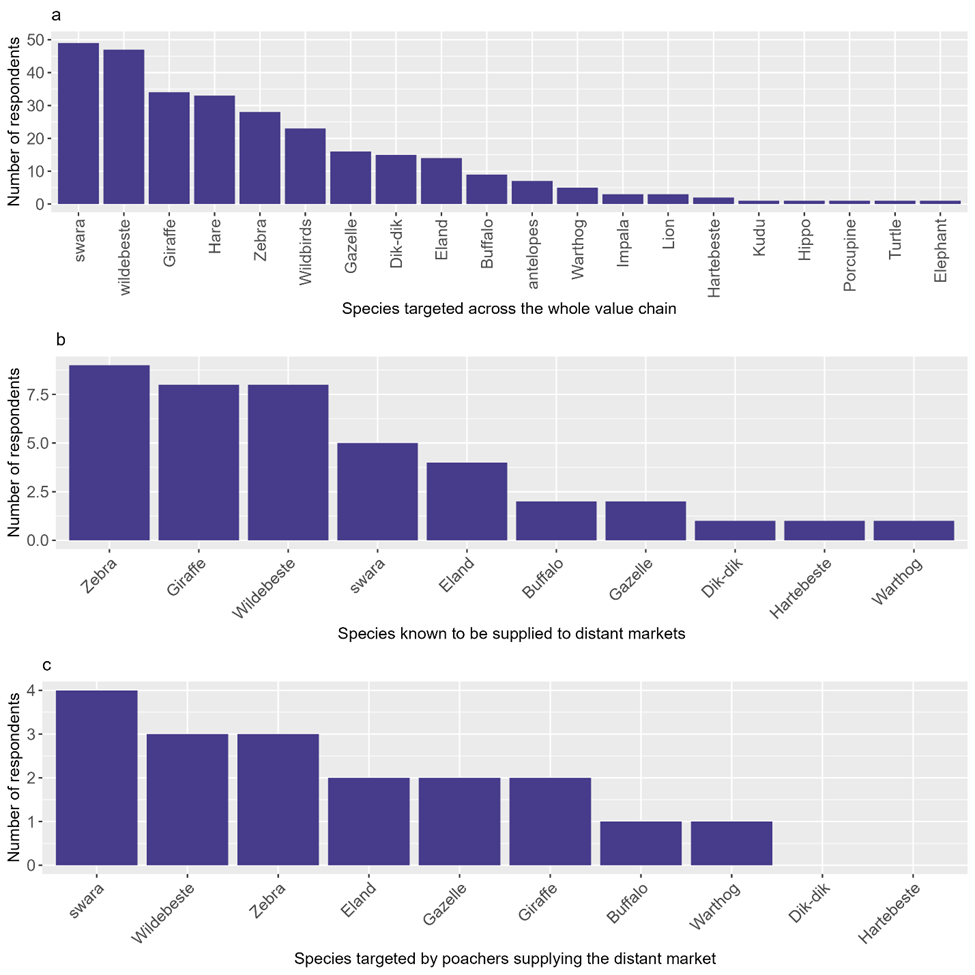


Figure 2: Frequency of wild animal species (a) respondent targeted along the value chain supplying the NMA; (b) that respondents knew were poached and supplied to the distant market; (c) that poachers hunted for wild meat they supplied to distant markets.
