# Supplementary material for: Limited knowledge of health risks along the illegal wild meat value chain in the Nairobi Metropolitan Area (NMA)
Source: PLoS One. 2025 Mar 26;20(3):e0316596. doi: 10.1371/journal.pone.0316596 (PMC11940438; doi:10.1371/journal.pone.0316596)
Supplement: S3 Fig — A graphical representation of the awareness levels for the specific diseases/pathogens, clinical signs, injuries, and the risk factors to infections that respondents were aware of as health risks attributable to wild meat. (DOCX) [file pone.0316596.s008.docx]

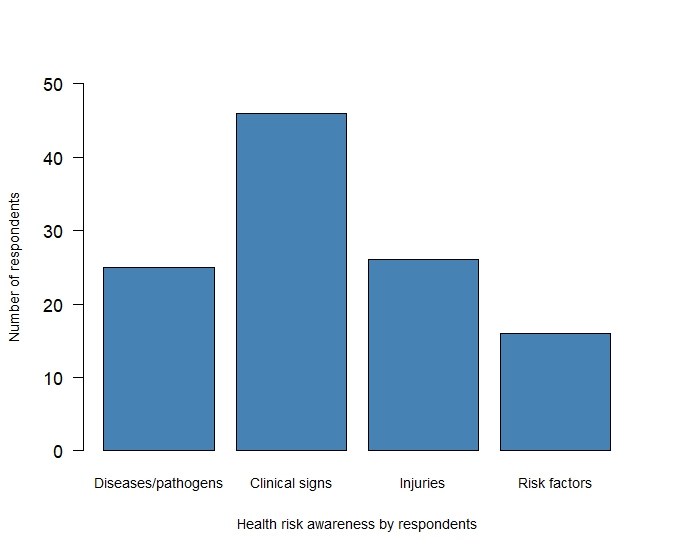


Figure 3: A graphical representation of respondent’s awareness levels for the specific diseases/pathogens, clinical signs, injuries, and the risk factors to infections attributable to wild meat.
