# Supplementary material for: Limited knowledge of health risks along the illegal wild meat value chain in the Nairobi Metropolitan Area (NMA)
Source: PLoS One. 2025 Mar 26;20(3):e0316596. doi: 10.1371/journal.pone.0316596 (PMC11940438; doi:10.1371/journal.pone.0316596)
Supplement: S1 Data — (PDF) [file pone.0316596.s009.pdf]

International Platform of Registered Systematic Review and Meta-analysis Protocols

INPLASY

Protocol for a systematic literature review on Knowledge of, attitude and practices towards meat hygiene and safety by meat handlers in the Nairobi Metropolitan Area (NMA)

INPLASY202460077
doi: 10.37766/inplasy2024.6.0077
Received: 20 June 2024
Published: 20 June 2024

Masudi, SP; Thomas, LT; Hassel, J; Pim, van H; Frank, van L; Otiende, MY; Ochieng, JW; Santangeli, A; Cook, AJE; Happi NA; Akpan, NS; Buij, R.

Corresponding author:
Sherril Phyllis

phyllis.masudi@wur.nl

Author Affiliation:
Wageningen University and Research.

ADMINISTRATIVE INFORMATION

Support - Funded by the Deutsche Gesellschaft für Internationale Zusammenarbeit GmbH (GIZ) through Wageningen University and Research with additional support from the German Federal Ministry for Economic Cooperation and Development through the One Health Research, Education and Outreach Centre in Africa (OHRECA).

Review Stage at time of this submission - Completed but not published.

Conflicts of interest - None declared.

INPLASY registration number: INPLASY202460077

Amendments - This protocol was registered with the International Platform of Registered Systematic Review and Meta-Analysis Protocols (INPLASY) on 20 June 2024 and was last updated on 20 June 2024.

INTRODUCTION

Review question / Objective We aimed to answer these questions from the systematic literature review: i) What food safety and hygiene practices do meat vendors in NMA engage in? ii) What knowledge, perceptions and attitudes do meat vendors in the NMA have about safe meat handling practices and the associated risks?

Rationale The legal restrictions on wild meat consumption and trade in Kenya has led to its covert harvesting, trade, and consumption . Wild meat thus infiltrates livestock meat markets, and it is suspected that the illegal trade targets unwitting end-users within the NMA . Based on our preliminary data, and findings from a previous study (Kimwele et al. 2012), wild meat is usually handled as and sold disguised as, or mixed with meat from livestock. Therefore, the practices, perceptions and attitudes meat vendors engage in when handling livestock meat would remain the same while handling wild meat . As such, understanding how meat vendors within the NMA handle livestock meat and other meat products they sell, their perceptions and attitudes towards meat handling practices and the associated health risks could serve as a proxy, and better guide to understanding the risk factors to their exposure to pathogens in the illegally sold wild meat. However, information on meat vendors practices, knowledge and perceptions in the NMA only exist as segregated data, and for different vendor categories and types of meat. This make inference to these data complicated. Pooling this information will therefore enable us collate and understand the probable risk factors based on practices and perception that would expose these vendors to

Masudi et al. INPLASY protocol 202460077. doi:10.37766/inplasy2024.6.0077 Downloaded from https://inplasy.com/inplasy-2024-6-0077/

pathogens in the wild meat that they could be fraudulently selling.

References

1. Kimwele CN, Karisa BK, Stokes M, Junga JO, Hanotte O, Skilton RA, McElroy D. DNA species surveillance: Monitoring bushmeat poaching and trading in Kenya using partial cytochrome b gene. African Journal of Biotechnology. 2012;11(78):14276-86.

Condition being studied We aimed to understand meat vendors practices, perceptions and practices towards safe meat handling and associated health risks in the NMA. Handling and consumption of animal products, like meat have been attributed to the emergence and reoccurrence of zoonotic outbreaks (Milbank et al. 2022, Pradhan et al. 2023). This situation is further alarming in countries where legal restrictions promotes informal animal sourced food systems where food safety is rarely adhered to (Delia et al. 2014). For instance in Kenya, consumptive use of wild animals, for food or as trade commodity is illegal. Consequently, there have been reports about infiltration of the livestock meat market in Kenya with bushmeat sourced from various poaching hotspots within the country (Kimwele et al. 2012, Otiende et al. 2023). To avoid the known legal implications from trading in bushmeat, meat vendors habitually sell wild meat disguised as, and sometimes mixed with livestock meat to unwitting customers. Vendors therefore do not handle wild meat any different from the other meats in the shop, despite the constant health concerns that have emerged regarding wild meat as the source of the next pandemic (Moloney et al. 2023, Milbank et al. 2022). Therefore, to understand their practices, knowledge and perceptions regarding the health risks from bushmeat in the NMA, we sort to use systematic literature review. We targeted the livestock meat trader node as the node where actors could be fraudulently handling and selling wild meat. This is an important node in the NMA wild meat value chain, yet one for which it is difficult to collect primary data via the key informant interviews we have previously used for the other value chain nodes (harvester node and consumption node at the community level). We attribute our reasoning to actors potentially refusing to participate out of fear of legal and economic ramifications. Therefore, by reviewing meat handling practices, perceptions and attitudes towards food safety and the associated risk amongst livestock meat vendors in the NMA, we will be able to have an idea on potential risk factors to exposure to pathogens that any vendor who could be fraudulently selling wild meat and their customers are exposed to.

References

1. Milbank C, Vira B. Wildmeat consumption and zoonotic spillover: contextualising disease emergence and policy responses. The Lancet Planetary Health. 2022 May 1;6(5):e439-48.
2. Pradhan AK, Karanth S. Zoonoses from animal meat and milk. InPresent knowledge in food safety 2023 Jan 1 (pp. 394-411). Academic Press.
3. Kimwele CN, Karisa BK, Stokes M, Junga JO, Hanotte O, Skilton RA, McElroy D. DNA species surveillance: Monitoring bushmeat poaching and trading in Kenya using partial cytochrome b gene. African Journal of Biotechnology. 2012;11(78):14276-86.
4. Otiende, M. Y., Muneza, A. B., Miyunga, A. A., Onyari, W., Fennessy, J., Omondi, P., Kariuki, L., Hassell, J., & Ochieng, J. W. Enhancing mapping of illegal wildlife trade hotspots in Kenya. 2023 (cited on 27/05/2024). Available on https://www.tourism.go.ke/wp-content/uploads/2023/09/WRTI-Sci-Con-Program-Booklet-Web-Version-2.pdf
5. Moloney GK, Gaubert P, Gryseels S, Verheyen E, Chaber AL. Investigating infectious organisms of public health concern associated with wild meat. Transboundary and Emerging Diseases. 2023 Aug 18;2023.
6. Tumelty L, Fa JE, Coad L, Friant S, Mbane J, Kamogne CT, Tata CY, Ickowitz A. A systematic mapping review of links between handling wild meat and zoonotic diseases. One Health. 2023 Oct 8:100637.
7. Grace D, Makita K, Kang'ethe E, Bonfoh B, Roesel K. Taking food safety to informal markets. InFood Safety and Informal Markets 2014 Oct 3 (pp. 11-22). Routledge.

METHODS

Search strategy The literature review was conducted as per the PRISMA-P guidelines for conducting systematic literature reviews. The search was conducted in the Web of Science, PubMed, and Google Scholar. We found it effective to use Google Scholar because it could yield any material related to the search including theses, project reports and presentation abstracts. It was important to consider these sources as most studies reporting on our review topic were commonly available in such forms. We acknowledge that some of these have not been peer reviewed and are only important at institutional level. We used the Boolean operators "AND" and "OR" to combine the relevant search terms. In Google Scholar, the following simple search term was used: Hygiene practices OR Food safety practices AND Meat AND Nairobi. In PubMed and Web of Science, we used the

Masudi et al. INPLASY protocol 202460077. doi:10.37766/inplasy2024.6.0077 Downloaded from https://inplasy.com/inplasy-2024-6-0077/

following syntax: ("Food safety practices" OR "Food hygiene practices") AND (knowledge OR awareness OR understanding) AND (attitude OR perception OR opinion) AND (meat handlers OR butcheries OR meat vendors OR meat markets) AND (Nairobi OR "Nairobi City" OR “Nairobi metropolitan area"). Because our study was only targeting a small area, and we anticipated only a few articles, only the lead author conducted the search.

Participant or population Our study targeted meat vendors in the NMA. We considered vendors working in informal premises such as makeshift stalls as seen with street vendors. We also considered vendors working in formally registered premises such hotels, catering units, or meat processing companies. We focused on meat vendors selling any meat and meat products that were raw or cooked, some processed while others were fresh. The study population considered vendors handling and selling meat from poultry, pigs, cattle, sheep, and goats and by products such as “Mutura” (African sausage made from intestine-encased minced meat, either mixed with blood, tripes, or seasonings, usually grilled).

Intervention Meat handling practices, perceptions and attitudes were the intervention measures we focused on. These included practices that vendors engaged in while handling meat, and those that could have an impact on both the safety of the vendor, and the meat sold at the shop. We considered hand hygiene, premise hygiene and sanitation, utensil hygiene, meat storage methods, compliance to PPE among others as some of the intervention indicators. We also sort to understand the context surrounding vendors practices by assessing their reported perceptions and attitudes. We considered reports on what they think drive their practices, in addition to their knowledge levels regarding safe meat handling and the associated risks when reported.

Comparator NA.

Study designs to be included Because we could only get a few articles from the search, we included all articles regardless of the study design. These included cross-sectional qualitative and quantitative studies.

Eligibility criteria We included studies conducted in the NMA, without language restriction. We included studies conducted from 2013 to 2023 and that which involved vendors who sold meat from any domestic animal as specified by the Kenyan law. We considered vendors selling both raw or processed meat. We included studies that focusing on either formal or informal sale of meat. In addition to the inclusion criteria , we excluded the following studies from the final inclusion;

1. Studies on food hygiene practices, knowledge and perception but are not focusing specifically on meat of any kind, and from any animal species;
2. Studies discussing other hygiene issues not directly relating to meat vendors and handler’s hygiene and food safety practices, attitude, and knowledge; and
3. Studies reporting on urban centers in Kenya, East Africa or developing countries but not specific to the NMA.

Information sources We considered electronic data bases including; Google Scholar; Web of Science; Pubmed.

Main outcome(s) Actors meat handling and other food safety practices as well as their knowledge and perceptions about such practices and the associated health risks.

Additional outcome(s) Drivers of meat handling and hygiene practices by the vendors in the NMA.

Data management Data was extracted from the retained articles by the lead author. Data were extracted deductively based on a predesigned thematic areas of meat hygiene and safety practices, knowledge, perceptions and attitudes and inductively for other emerging but relevant study outcomes. All extracted information were collated in an excel sheet prior to using them for descriptive thematic analysis to provide a summary of the review outcome.

Quality assessment / Risk of bias analysis Based on the Newcastle-Ottawa scale (Modesti et al. 2016), individual study quality was assessed using the following parameters as quality indicators; (i) selection criteria for study population, (ii)study comparability and (iii)assessment of the study outcomes based on the reported statistical analysis methods. For qualitative studies, an asterisk (*) was awarded when we thought that the analysis protocol was satisfactorily described, as per a previous protocol by Alarcon et al. 2017. Only the lead author conducted quality assessment of the studies included.

References

1. Modesti PA, Reboldi G, Cappuccio FP, Agyemang C, Remuzzi G, Rapi S, Perruolo E, Parati G, ESH Working Group on CV Risk in Low Resource Settings. Panethnic differences in blood pressure in Europe: a systematic review and meta-analysis. PloS one. 2016 Jan 25;11(1):e0147601.

Masudi et al. INPLASY protocol 202460077. doi:10.37766/inplasy2024.6.0077 Downloaded from https://inplasy.com/inplasy-2024-6-0077/

2. Alarcon P, Fèvre EM, Murungi MK, Muinde P, Akoko J, Dominguez-Salas P, Kiambi S, Ahmed S, Häsler B, Rushton J. Mapping of beef, sheep and goat food systems in Nairobi—A framework for policy making and the identification of structural vulnerabilities and deficiencies. Agricultural systems. 2017 Mar 1;152:1-7.

Strategy of data synthesis We considered a thematic qualitative analysis for the extracted review outcome.

Subgroup analysis Not conducted.

Sensitivity analysis Not conducted.

Language restriction No restriction imposed.

Country(ies) involved Kenya, Netherlands, Nigeria, USA, UK, Finland.

Keywords Hygiene, safety, meat, handling, vendors, food, Nairobi Metropolitan Area.

Dissemination plans The results of this review will be published, together with another set of data from key informant interviews targeting other actors along the wild meat value chain supplying the NMA, in a peer reviewed journal. We will also disseminate our findings to targeted communities and stakeholders in the government, Nairobi County's Public Health Department, the Kenya Wildlife Services to foster multisectoral response to our study recommendations.

Contributions of each author

Author 1 - Sherril P. Masudi - Study conception, design, implementation, data analysis and manuscript writing.
Email: phyllissherril@gmail.com
Author 2 - Lian F Thomas - Study conception, design, implementation, data analysis and manuscript review.
Email: l.thomas@cgiar.org
Author 3 - James Hassel - Study conception, design, implementation, and manuscript review.
Email: hasselljm@si.edu
Author 4 - Pim van Hooft - Study conception, design, implementation, and manuscript review.
Email: pim.vanhooft@wur.nl
Author 5 - Frank van Langevelde - Study conception, design, implementation, and manuscript review.
Email: frank.vanlangevelde@wur.nl
Author 6 - Moses Y. Otiende - Study conception, design, implementation, and manuscript review.
Email: moses.yongo@gmail.com
Author 7 - Joel W. Ochieng' - Study conception, design, implementation, and manuscript review.
Email: jochieng@uonbi.ac.ke
Author 8 - Andrea Santangeli - Study conception, design, implementation, and manuscript review.
Email: andrea.santangeli@helsinki.fr
Author 9 - Annie J. E. Cook - Study conception, design, implementation, and manuscript review.
Email: e.cook@cgiar.org
Author 10 - Anise Happi - Manuscript review.
Email: happia@run.edu.ng
Author 11 - Samuel Akpan - Manuscript review
Email: samuel.akpan@wur.nl
Author 12 - Ralph Buij - Study conception, design, implementation, and manuscript review.
Email: ralph.buij@wur.nl

Masudi et al. INPLASY protocol 202460077. doi:10.37766/inplasy2024.6.0077 Downloaded from https://inplasy.com/inplasy-2024-6-0077/
